# Interaction of common variants of FTO gene and Dietary Inflammatory Index on obesity measures: Tehran Lipid and Glucose Study

**DOI:** 10.1136/bmjnph-2023-000665

**Published:** 2023-11-23

**Authors:** Negin Haji-Hosseini-Gazestani, Firoozeh Hosseini-Esfahani, Asal Ataie-Jafari, Golnoosh Goodarzi, Maryam S Daneshpour, Parvin Mirmiran, Fereidoun Azizi

**Affiliations:** 1 Nutrition and Endocrine Research Center, Research Institute for Endocrine Sciences, Shahid Beheshti University of Medical Sciences, Tehran, Iran; 2 Nutrition and Endocrine Research Center, Shahid Beheshti University of Medical Sciences Research Institute for Endocrine Sciences, Tehran, Iran; 3 Department of Nutrition, Science and Research Branch, Islamic Azad University, Tehran, Iran; 4 Cellular and Molecular Endocrine Research Center, Research Institute for Endocrine Sciences, Shahid Beheshti University of Medical Sciences, Tehran, Iran; 5 Endocrine Research Center, Research Institute for Endocrine Sciences, Shahid Beheshti University of Medical Sciences, Tehran, Iran

**Keywords:** Weight management, Dietary patterns, Nutrition assessment

## Abstract

**Background:**

This study aimed to examine the interaction of Dietary Inflammatory Index (DII) and fat mass and obesity-associated gene (FTO) single-nucleotide polymorphisms (SNPs) on change in obesity measures.

**Methods:**

A total of 4480 participants from the Tehran Lipid and Glucose Study were selected. DII was calculated using a Food Frequency Questionnaire. The FTO SNPs rs8050136, rs14211085 and rs1121980 were selected. Changes in obesity measures were calculated.

**Results:**

In individuals with risk allele of FTO SNP rs8050136, greater adherence to DII was associated with increased odds of higher waist circumference (WC) (OR, Q1–Q4: 1, 0.87, 0.88, 0.94; P trend=0.01), but deceased odds of waist to hip ratio (WHR) (OR, Q1–Q4: 1, 0.85, 0.76, 0.70; P trend=0.01). Moreover, higher score of DII was significantly related to elevated odds of having high Visceral Adiposity Index (VAI) in individuals with wild-type genotype of FTO SNPs. For changes in WC, a significant interaction was identified between FTO rs1421085 and DII; the second quartile of DII was associated with increased odds of having a high WC in carriers of wild variant (TT genotype) of rs1421085 (OR 1.43; 95% CI 1.04 to 1.97), but not in individuals with risk allele of this SNP (TC CC). Although there are significant relationships between SNPs or genetic risk score and change in WHR or VAI, but there is no significant interaction between FTO SNPs and DII regarding change in body mass index, WHR and VAI.

**Conclusions:**

There may be an interactive effect between DII and the FTO rs1421085 genotypes on change in WC.

**WHAT IS ALREADY KNOWN ON THIS TOPIC**

- Some foods have proinflammatory effects, which may induce obesity.
- Some variants of fat mass and obesity-associated gene (FTO) gene are associated with the inflammatory marker C reactive protein (CRP), and CRP has been directly correlated with body mass index.

**WHAT THIS STUDY ADDS**

- Greater Dietary Inflammatory Index (DII) adherence was associated with increased risk of higher waist circumference in wild type of rs14211085.
- Higher score of DII was significantly related to elevated odds of having high VAI in individuals with wild-type genotype of FTO single nucleotide polymorphisms.

**HOW THIS STUDY MIGHT AFFECT RESEARCH, PRACTICE OR POLICY**

- Considering gene–diet interactions can deliver recommendations for better prevention of obesity.

## Introduction

Obesity was defined as an excessive accumulation of adipocytes. It is a worldwide epidemic with increasing prevalence, affecting 1.9 billion people globally.^1^ It is not only related to major chronic diseases such as diabetes, cardiovascular diseases, metabolic syndrome, malignancies, subclinical inflammation and premature death^2^ but also has unfavourable effects on quality of life and individuals’ self-esteem.^3^ Thus, appropriate prevention of obesity is critical to reduce its adverse health-related consequences.

Obesity is a multicausal problem, influenced by the interaction between genes and various environmental factors, especially diet, sleep deprivation, physical inactivity and even gut microbiota.^4^ Obesity is also featured by a state of chronic systemic low-grade inflammation, a condition proposed to underlie its association with chronic diseases.^5^ However, a bidirectional relationship has been identified between inflammation and obesity, so that inflammation may play a role in the progression of obesity.^6^ Recently, the modulation of chronic inflammation by diet has received significant attention. Evidence suggests that higher consumption of foods containing antioxidant compounds such as vegetables, fruits and whole grains are related to decreasing in inflammation; while, processed meats, refined carbohydrates, saturated fatty acids (SFAs) and trans fatty acids has proinflammatory effects.^7^ The study of each foods/nutrients involved in inflammation individually cannot reveal the overall potential for dietary inflammation, so the Dietary Inflammatory Index (DII) has been recently developed to specifically quantify the inflammatory potential of diet by considering potential synergistic or antagonistic effects of inflammatory compounds in the diet. Previous studies reported that DII has a close association with inflammatory biomarker levels like C reactive protein (CRP).^8^

Studies examining the relationship between DII and measures of obesity, including body mass index (BMI), waist circumference (WC) and waist-hip ratio (WHR) have yielded contradictory findings, with positive^9^ or null^10^ associations. Differences in genetic background and gene–diet interactions may partially explain these inconsistences.^11^ It has been estimated that genetic background predicts 65%–80% of susceptibility to obesity.^12^ Genome-wide association studies (GWAS) have identified some genetic variants related to obesity, of which, fat mass and obesity-associated gene (FTO) is reported to be the strongest one.^13^ Studies have found that FTO single-nucleotide polymorphisms (SNPs) rs1121980, rs1421085 and s8050136 are associated with main measures of adiposity such as BMI, WC and WHR.^14^ Moreover, rs9939609 variants of FTO gene are associated with the inflammatory marker CRP, and CRP has been directly correlated with BMI,^15^ hypothesising a possible interaction of FTO gene and DII on obesity. In the previous studies, FTO gene had an interaction with dietary patterns concerning obesity phenotypes, but its interaction with DII is not elucidated yet.^16^ This study aimed to assess the interaction of common variants of FTO gene, individually or in combination by genetic risk score (GRS), and DII on measures of obesity in a group of Tehranin adult population.

## Material and methods

### Study population

This study was conducted on participants of the Tehran Lipid and Glucose Study (TLGS), a population-based prospective investigation designed in 1999 in Tehran, Iran to examine risk factors of non-communicable diseases.^17^ The first assessment of the TLGS was performed from 1999 to 2001, on 15 005 subjects with age ≥3 years, and follow-up measurements were performed every 3 years (2002–2005; 2005–2008; 2008–2011 and 2011–2014). A total of 10 086 individuals participated in the fourth phase of the TLGS during 2008–2011. After excluding subjects aged ≤18 years old (n=1243), subjects taking antiobesity drugs and those with missing data on dietary intake and anthropometric indices (n=1961), a total of 6882 participants were recruited as the initial population of this study and were followed up for obesity phenotypes until the next survey of the study (2011–2014).

Subjects were excluded because of the lack of DNA samples or DNA purification data out of the range of 1.7<A260/A280<2 (n=1600), reporting unusual food intake (energy intake less than 800 or more than 4200 kcal/day) (n=637), or being pregnant (n=80) or lactating (n=85). Finally, a total of 4480 participants were entered in the current study.

## Dietary assessment

Dietary intakes of all subjects were measured using a valid and reliable 168-item Food Frequency Questionnaire (FFQ).^18^ Expert nutritionists asked subjects to report their consumption frequency of each food item over the previous year on a daily, weekly and monthly basis through face-to-face interview. Then portion sizes assessed by household measures for each food were converted into grams. Finally, energy and nutrient contents of diet was estimated with the use of the United States Department of Agriculture (USDA) food composition table. For local foods that were unavailable in USDA, the Iranian food composition table was used.

## Calculation of DII

Based on the approach of Shivappa *et al*,^19^ the DII score was calculated using 37 dietary parameters extracted from the FFQ questionnaire, including; energy, total carbohydrates, vitamin A, total fat, total protein, trans fat, SFA, folic acid, polyunsaturated fat (PUFA), monounsaturated fat (MUFA), omega 3, omega 6, fibre, riboflavin, cholesterol, niacin, vitamin D, thiamin, vitamin C, vitamin E, vitamin B_12_, vitamin B_6_, magnesium, iron, selenium, zinc, caffeine, tea, beta-carotene, isoflavon, flavon, flavanone, flavonol, anthocyanidin, garlic, onion and pepper. Energy-adjusted intakes of dietary parameters were used to calculate DII. In order to compute the DII score, the mean intake of each dietary parameter was standardised by subtracting the mean global intake of dietary parameters from the real individual’s consumption and dividing it by the global SD to create a z-score. Then, the z-scores were converted to proportions and centred by multiplying the amounts by 2 and subtracting 1 to avoid skewness and to normalise the scoring system. Afterward, the centred percentile amounts of dietary parameters were multiplied by the dietary parameter-specific inflammatory impact scores to yield the dietary parameter-specific DII scores.^20^ The validity of DII score has been reported based on 24-hour recall and FFQ questionnaire.^21^ Scores of all food items/nutrients were summed up to calculate the overall DII score of diet for each subject. Higher DII score represents a more inflammatory potential of diet, whereas lower DII score shows a more anti-inflammatory diet.

## DNA extraction and genotyping

We selected 17 available potentially functional and common SNPs of FTO gene from list of GWAS catalogue SNPs based on minor allele frequency ˃0.2 and p ˂10^−7^. Among the FTO gene SNPs, three available polymorphisms (rs8050136, rs1121980 and rs1421085) had a strong correlation (r^2^>0.8) with the other SNPs and moderate correlation (r^2^<0.7) with each other.

There is a high (r^2^=1) linkage disequilibrium between rs9939609 and the three SNPs (rs8050136, rs3751812 and rs17817449) according to data from South and East Asians, hence rs9939609 was not engaged in the GRS analysis and calculation.^22 23^ Also, due to limited data accessible, the most associated SNPs with dietary intake and obesity were chosen.

DNA was extracted from peripheral blood samples using proteinase K and salting out procedure. The concentration and purity of the extracted DNA was then examined by NanoDrop spectrophotometer (Thermo Scientific Company, USA). Samples with the ratio of the absorbance at 260 and 280 nm out of the range of 1.7–2 were removed due to low quality and concentration. The extracted DNA was stored at –80°C.

Portions of DNA samples were genotyped with HumanOmniExpress-24-v1-0 bead chips, containing 649 932 SNP loci, with an average mean distance of 4 kb at the deCODE genetics company (Reykjavik, Iceland), according to the manufacturer’s specifications (Illumina, San Diego, California, USA). For quality control procedures, PLINK program (V.1.07) and R statistic (V.3.2) were used, with the total genotyping rate of 0.9774. After quality control procedures, the genotyping data of FTO polymorphisms were used for data analysis.

## Measures of obesity

Weight of all subjects was assessed to the nearest 100 g by a digital scale (model 707, range 0.1–150 kg; Seca, Hamburg, Germany); while they were lightly clothed, and not wearing shoes. Height was measured with a precision of 5 mm, with the use of a standard tape, without shoes in a standing position. BMI was calculated as weight (kg)/height squared (m^2^). WC was measured at the level of umbilicus over light clothing at the end of a normal expiration using a flexible tape to the nearest 0.1 cm, without exerting any pressure on the body surface. Hip circumference (HC) was measured at the widest point over the buttocks using a standard tape metre. Waist to height ratio (WHR) was also calculated as WC (cm)/height (cm). Visceral Adipose Index (VAI) was calculated using the following formula,^24^ which is gender-specific:

$$MalesVAI=\left(\frac{WC}{39.68+\left(1.88*BMI\right)}\right)*\left(\frac{TG}{1.03}\right)*\left(\frac{1.31}{HDL}\right)$$

$$FemalesVAI=\left(\frac{WC}{36.58+\left(1.89*BMI\right)}\right)*\left(\frac{TG}{0.81}\right)*\left(\frac{1.52}{HDL-C}\right)$$

In which WC is waist circumference, TG is triglyceride, and HDL-C is high-density lipopr

otein cholesterol.

Anthropometric changes were computed by subtracting the values at baseline from the corresponding values at follow up-measurements; an increase in VAI, WC, BMI or WHR was considered as anthropometric change being positive or >0.

## General characteristics and physical activity

General information of participants including sex, age, smoking, medication use, medical history and education level was obtained using a self-administered questionnaire. Physical activity was evaluated by using a modified physical activity questionnaire validated for TLGS^25^; physical activity was expressed as metabolic equivalent/minute/week (MET/min/wk).

## Obesity GRS calculation

The weighted methods were applied to compute GRS according to the three selected polymorphisms.^26^ Based on the BMI elevating risk alleles, the scores of 0, 1 or 2 were given to the polymorphisms. After that, each SNP was weighted by its relative effect sizes (OR), which were based on the coefficients resulted from the logistic regression analysis conducted on the population of the study. To compute the GRS, the following equation was used: GRS=(OR1×SNP1 + OR2 × SNP2 + … + OR_n*SNP*n)._

Where odds ratio (OR) is the odds of each SNP on BMI.^27^

## Statistical analysis

All statistical analyses were done using the SPSS V.21 software (SPSS) Quantitative and categorical variables were reported respectively as mean±SD and percentages. The statistically significant level was set at p˂0.05. Comparing qualitative and quantitative variables across quartiles of DII and FTO genotypes was performed using the χ^2^ and analysis of variance tests, respectively. Deviation from Hardy-Weinberg equilibrium was also tested by χ^2^ test. Logistic regression analysis was performed to obtain the OR and corresponding 95% CI of increased obesity indices associated with DII quartiles and genotypes of FTO gene polymorphisms. The results were adjusted for potential confounders including age, sex, energy intake, education level (>14 and ≤14 years), physical activity (low, moderate and high) and smoking (current, ex-smoker or never smoked). Moreover, interactions between DII and FTO gene SNPs in relation to changes in obesity indices (WC, WHR, BMI and VAT) were evaluated using logistic regression analysis. Participants were divided into eight groups, according to the combined role of quartiles of DII and dominant model of FTO SNP genotypes in estimating OR for outcomes; the lowest quartile of DII and homozygote genotype of major allele was used as the reference group. Changes in WC, WHR, BMI and VAI were categorised as ˃0 and ≤0, in which changes ˃0 were coded as 1 and changes ≤0 were coded as 0 in the logistic models. Linear regression analysis was used for the association of anthropometric indices at baseline with DII score based on FTO SNP genotypes. P interaction was calculated by using general linear model.

## Results

Table 1 presents the characteristics and nutrient intakes of individuals across quartiles of DII. Participants with higher quartiles of DII were younger, had lower physical activity and lower baseline values for all obesity measures, including BMI, WC, WHR and VAI, compared with the lower quartiles (p<0.001), but no significant difference in changes in obesity measures was found across quartiles of DII. Moreover, individuals with higher score for DII, compared with those with lower score, had significantly higher intakes of total fat, SFA, and MUFA and lower intakes of energy, carbohydrate, total fibre, protein, trans fat and PUFA (p<0.001).

**Table 1:** Characteristics and dietary intake of participants according to quartiles of the Dietary Inflammatory Index (DII) among adult participants of the Tehran Lipid and Glucose Study

Characteristics	Quartiles of the DII				
	Q1 (n=939)	Q2 (n=954)	Q3 (n=951)	Q4 (n=985)	P value
Median	6.24	6.70	6.99	7.27	0.28
Age (years)	43.2±13.8	41.7±13.7	39.2±13.5	37.4±13.5	<0.001
Smoking (%)	19.9	26.4	25	28.7	<0.001
Sex (% men)	32.8	38.8	49.4	61.2	<0.001
Education level*	24.8	24.8	25.0	25.4	0.74
Physical activity (MET/min/week)	744±820	705±829	643±747	589±600	<0.001
Anthropometry					
Baseline BMI (kg/m^2^)	27.8±4.68	27.4±4.60	26.8±4.69	26.4±4.87	<0.001
Delta BMI (Kg/m^2^)	0.44±2.17	0.32±1.96	0.40±2.00	0.44±1.95	0.54
Baseline WC (cm)	93.5±11.9	92.7±11.8	91.2±12.2	89.9±12.4	<0.001
Delta WC (cm)	0.35±5.87	0.47±6.31	0.73±6.14	0.74±6.04	0.40
Baseline WHR	0.92±0.08	0.92±0.08	0.91±0.08	0.90±0.08	<0.001
Delta WHR	0.01±0.30	0.00±0.04	0.00±0.04	0.00±0.05	0.29
Baseline VAI	2.44±1.96	2.32±2.02	2.25±1.65	2.06±1.58	<0.001
Delta VAI	−0.01±0.14	−0.00±0.12	−0.00±0.12	−0.00±0.15	0.19
Dietary intakes					
Energy intake (kcal/day)	2961±636	2523±604	2231±587	1910±58	<0.001
Carbohydrate (% of energy)	61.7±6.83	59.3±6.12	58.1±5.85	56.1±6.42	<0.001
Total fibre intake (gr/1000 Kcal)	13.0±4.24	10.5±2.58	9.19±2.21	7.50±2.04	<0.001
Protein intake (% of energy)	15.3±3.89	15.0±3.45	14.6±2.74	14.4±3.68	<0.001
Total fat (% of energy)	28.1±5.64	29.3±5.90	30.2±5.82	31.7±6.63	<0.001
Trans fatty-acids (gr/day)	6.61±4.92	6.03+4.12	5.82±4.15	5.57±4.51	<0.001
Saturated fat (% of energy)	8.58±2.50	9.27±2.20	9.94±2.38	11.1±3.02	<0.001
MUFA (% of energy)	9.54±3.10	9.79±2.88	10.1±2.53	10.4±2.56	<0.001
PUFA (g)	19.3±7.74	16.7±7.16	15.1±6.59	12.8±6.21	<0.001

Continuous variables were reported as means±SDs (using ANOVA test).

Categorical variables were analysed using the χ2 test and reported as a percentage (%).

*Educational level≥14 years.

ANOVA, analysis of variance; BMI, body mass index; DII, Dietary Inflammatory Index; MET/min/week, metabolic equivalent minutes per week; MUFA, monounsaturated fatty acid; PUFA, polyunsaturated fatty acid; VAI, Visceral Adiposity Index; WC, waist circumference; WHR, waist-to-hip ratio.

Adjusted ORs with corresponding 95% CIs for measures of obesity as dichotomous outcomes according to quartiles of DII, FTO SNP genotypes and GRS are presented in tables 2 and 3. There was an increasing trend for the risk of having a high WC in individuals with GA+AA genotypes of rs8050136 across quartiles of DII (P trend=0.01). FTO rs1421085 and DII had a significant interaction in relation to WC (P interaction=0.03); the second quartile of DII was associated with a 1.43-fold (OR 1.43, 95% CI 1.04 to 1.97) odds of having a high WC in subjects with TT genotype of rs1421085 compared with individuals with TC+CC genotype (table 2).

**Table 2:** Adjusted ORs (95% CI) for changes in BMI and WC according to quartiles of Dietary Inflammatory Index score and FTO SNP genotypes

	Change in BMI*				P trend	P interaction	Change in WC*				P trend	P interaction
	Q1	Q2	Q3	Q4			Q1	Q2	Q3	Q4		
rs1121980						0.76						0.38
CC	1	1.07 (0.77–1.48)	0.97 (0.69–1.38)	0.94 (0.64–1.39)	0.75		1	1.44 (1.04–2.00)	1.33 (0.94–1.88)	1.32 (0.90–1.94)	0.20	
CT+TT	1	0.84 (0.66–1.03)	0.79 (0.61–1.07)	0.81 (0.61–1.07)	0.10		1	0.96 (0.75–1.21)	0.94 (0.73–1.21)	0.98 (0.74–1.29)	0.08	
rs1421085						0.41						0.03
TT	1	1.11 (0.81–1.53)	0.99 (0.70–1.39)	0.89 (0.61–1.31)	0.60		1	1.43 (1.04–1.97)	1.33 (0.95–1.87)	1.37 (0.94–2.00)	0.10	
TC+CC	1	0.80 (0.64–1.02)	0.76 (0.58–0.99)	0.82 (0.61–1.09)	0.10		1	0.94 (0.74–1.19)	0.94 (0.73–1.21)	0.97 (0.73–1.28)	0.05	
rs8050136						0.42						0.30
GG	1	1.15 (0.85–1.56)	0.94 (0.68–1.31)	0.87 (0.60–1.25)	0.43		1	1.56 (1.15–2.10)	1.43 (1.04–1.97)	1.32 (0.92–1.89)	0.05	
GA+AA	1	0.77 (0.60–0.99)	0.78 (0.59–1.02)	0.81 (0.60–1.09)	0.13		1	0.87 (0.68–1.12)	0.88 (0.67–1.15)	0.94 (0.71–1.25)	0.01	
GRS						0.47						0.05
GRS<2.83	1	1.14 (0.84–1.54)	0.95 (0.69–1.32)	0.84 (0.58–1.22)	0.38		1	1.50 (1.11–2.03)	1.31 (0.94–1.81)	1.22 (0.84–1.76)	0.23	
GRS≥2.83	1	0.77 (0.60–1.00)	0.77 (0.59–1.02)	0.83 (0.61–1.11)	0.16		1	0.82 (0.64–1.06)	0.83 (0.63–1.09)	0.88 (0.65–1.17)	0.32	

ORs (95% CI) were calculated using logistic regression model, adjusted for education level, age, gender, smoking status, physical activity and energy intake. Participants were classified (eight groups) according to quartiles of Dietary Inflammatory Index and dominant model of FTO polymorphism genotypes or GRS≥median and <median. The lowest quartile of Dietary Inflammatory Index score and homozygote genotype of major allele was used as the reference group.

*BMI or waist circumference (WC) change was calculated by subtracting the BMI or WC at baseline, from their measurements over a mean of 3 years follow-up; an increase in BMI or WC was defined if BMI or WC change was positive or >0.

BMI, body mass index; FTO, fat mass and obesity-associated gene; GRS, genetic risk score; Q, quartiles of dietary inflammatory index; SNP, single-nucleotide polymorphism.

**Table 3:** Adjusted ORs (95%confidence interval) for changes in waist-to-hip ratio and Visceral Adiposity Index according to quartiles of Dietary Inflammatory Index score and FTO SNP genotypes

	Change in WHR*				P trend	P interaction	Change in VAI*				P trend	P interaction
	Q1	Q2	Q3	Q4			Q1	Q2	Q3	Q4		
rs1121980						0.48						0.31
CC	1	1.36 (0.97–1.91)	1.36 (0.95–1.95)	1.24 (0.83–1.86)	0.20		1	1.47 (1.07–2.03)	1.41 (1.00–1.98)	1.53 (1.04–2.24)	0.02	
CT+TT	1	0.97 (0.75–1.24)	0.86 (0.66–1.13)	0.78 (0.58–1.03)	0.08		1	1.05 (0.83–1.33)	1.01 (0.79–1.31)	0.96 (0.73–1.27)	0.82	
rs1421085						0.58						0.27
TT	1	1.34 (0.97–1.87)	1.40 (0.98–1.99)	1.33 (0.90–1.97)	0.10		1	1.52 (1.11–2.08)	1.39 (1.00–1.94)	1.63 (1.12–2.37)	0.01	
TC+CC	1	0.95 (0.74–1.22)	0.85 (0.65–1.11)	0.75 (0.56–1.01)	0.05		1	1.01 (0.80–1.29)	1.01 (0.78–1.31)	0.93 (0.70–1.23)	0.71	
rs8050136						0.06						0.14
GG	1	1.53 (1.12–2.09)	1.58 (1.13–2.21)	1.34 (0.92–1.95)	0.05		1	1.54 (1.14–2.07)	1.47 (1.06–2.02)	1.54 (1.08–2.20)	0.01	
GA+AA	1	0.85 (0.66–1.11)	0.76 (0.58–1.00)	0.70 (0.52–0.95)	0.01		1	0.99(0.77–0.27)	0.96 (0.74–1.25)	0.91 (0.68–1.21)	0.55	
GRS						0.10						0.22
GRS<2.83	1	1.53 (1.12–2.09)	1.50 (1.07–2.09)	1.33 (0.91–1.94)	0.07		1	1.49 (1.11–2.00)	1.40 (1.01–1.92)	1.52 (1.06–2.17)	0.02	
GRS≥2.83	1	0.84 (0.65–1.09)	0.75 (0.57–0.99)	0.70 (0.52–0.95)	0.01		1	0.99 (0.77–1.27)	0.97 (0.75–1.27)	0.92 (0.69–1.23)	0.64	

ORs (95% CI) were calculated using conditional logistic regression model, adjusted for education level, age, gender, smoking status, physical activity and energy intake. Participants were classified (eight groups) according to quartiles of Dietary Inflammatory Index and dominant model of FTO polymorphism genotypes or genetic risk score (GRS) ≥median and <median. The lowest quartile of Dietary Inflammatory Index score and homozygote genotype of major allele was used as the reference group.

*Waist-to-hip ratio (WHR) and Visceral Adiposity Index (VAI) change was calculated by subtracting the WHR or VAI at baseline, from their measurements over a mean of 3 years follow-up; an increase in WHR or VAI was defined if WHR or VAI change was positive or >0.

FTO, fat mass and obesity-associated gene; Q, quartiles of dietary inflammatory index; SNP, single-nucleotide polymorphism.

Higher score for DII, compared with lower scores, was significantly associated with lower odds of high WHR in individuals with a higher GRS (OR 0.70, 95% CI 0.52 to 0.95, P trend=0.01) and in carriers of GA+AA genotypes of rs8050136 (OR 0.70, 95% CI 0.52 to 0.95, P trend=0.01) (table 3). In contrast, higher score for DII, compared with lower scores, was significantly related to elevated odds of having high VAI in individuals with a lower GRS (OR 1.52, 95% CI 1.06 to 2.17, P trend=0.02) and in carriers of wild-type genotype of rs1121980 (OR 1.53, 95% CI 1.04 to 2.24, P trend=0.02), rs1421085 (OR 1.63, 95% CI 1.12 to 2.20, P trend=0.01) and rs8050136 (OR 1.54, 95% CI 1.08 to 2.17, P trend=0.02) (table 3). No significant interaction was revealed between FTO SNPs and DII, regarding change in general obesity, WHR and VAI.

The DII score was inversely associated with baseline BMI, WC, WHR and VAI in minor allele carriers of three FTO SNPs and in individuals with high GRS (online supplemental table 1). The interaction effects of FTO SNPs and DII score in relation to baseline BMI and WC were significant. The interaction effects of FTO SNPs and DII score in relation to baseline WHR and VAI were not significant.

10.1136/bmjnph-2023-000665.supp1 Supplementary data

## Discussion

The present cohort study explored the interaction of DII with FTO gene SNPs in relation to obesity indices.The results indicated that, in individuals with risk allele of FTO SNP rs8050136 (carriers of GA+AA genotypes), greater adherence to DII was associated with increased odds of higher change in WC, but decreased odds of change in WHR. Moreover, higher score for DII was significantly related to elevated odds of having high VAI in individuals with wild-type genotype of all investigated SNPs. For change in WC, a significant interaction was identified between FTO rs1421085 and DII, so that the second quartile of DII was associated with increased odds of having a high WC in carriers of wild variant (TT genotype) of rs1421085, but not in individuals with risk allele of this SNP (TC+CC genotype). Although there are significant relationships between SNPs or GRS and change in WHR or VAI, but there is no significant interaction between FTO SNPs and DII regarding change in BMI, WHR and VAI.

Obesity is resulted from a complex interaction between genetic background and environmental factors, including low physical activity and unhealthy dietary patterns.^24 28^ It has been suggested that diet may affect obesity by increasing chronic low-grade inflammatory status.^29^ Accumulating evidence, in different populations, has linked a variety of circulating markers of inflammation such as IL-6, TNF-a, CRP and total leucocyte count to measures of obesity.^30^ CRP is reported to be related to BMI and hip and WCs.^31^ In a Mediterranean population,^32^ a proinflammatory diet was positively related to general and abdominal obesity, independent of recognised risk factors of obesity such as physical activity, daily energy consumption, marital status, educational level, age, diabetes, hypertension and smoking status; the study by Ruiz-Canela *et al* 9 supports that diet might play a role in obesity, especially central obesity, via inflammatory modulation mechanisms. The nature of inflammatory status in obesity is not well-identified. Inflammation is demonstrated to be induced by adipose tissue, but this link seems to be bidirectional, therefore, making a defective cycle, since excess energy intake and several particular food parameters also have been related to inflammation.^33^ Mechanistically, a diet with a high inflammatory potential can activate pathogen-related molecular patterns, such as Nod-like receptors and Toll-like receptors, which induce inflammation in the adipose tissue.^9^ Furthermore, a diet with a high DII has important effects on the gut microbiota, playing a key role in low-grade inflammatory status related to adiposity.^34^ The mechanisms, through which a diet with high DII may lead to obesity, remain uncertain. Inflammatory cytokines, such as TNF-a, IL-6 and IL-1 could induce appetite, by increasing fat deposition and energy intake.^35^ Also, in chronic inflammatory condition, adiposity signals including insulin and leptin stimulate the peripheral sympathetic nervous system, which subsequently induces b-adrenegic desensitisation, resulting in an increase in body fat.^5 36^ Another potential explanation is that excess intake of some nutrients can trigger inflammation in hypothalamus, as a potential cause of obesity.^9^

This study identified that the association of DII with indices of central obesity is modified by FTO SNPs; but, no significant difference in BMI was found across categories of DII and FTO SNPs. The relation of FTO SNPs (rs8050136) to BMI in Asian populations has been inconsistent^37^; these inconsistencies might be elucidated by differences in sex or age of participants, definition used for obesity, the impacts of environmental variables as covariates, and the dissimilar allelic frequencies reported in racial groups. Furthermore, the link of FTO SNPs to BMI is reported to be age-dependent, strengthening with age up to a peak at age 20 years, and then weakening with increasing age.^38^ Therefore, the lack of relationship observed in this study may result from the age of the participants (40.31 years). The mechanism responsible for the effect of FTO on obesity is not well known. It is reported that FTO gene affects adiposity by regulating energy intake, energy expenditure and fat distribution.^24^ Also, FTO expression affects levels of leptin, perilipin and visfatin in adipose tissue, which are involved in various pathways of metabolism.^39^ The interaction of the DII and FTO SNP on WC has important clinical utility to develop personalised preventive approach for obesity based on genetic context of people that is adapted to meet people’s needs.

To the best of our knowledge, this is the first study to recognise an interaction between DII and FTO rs1421085 for the susceptibility to increased WC. The chief strengths of the current study are: the prospective design of the investigation, a large sample size that increases the statistical power, adjustment analyses for potential covariates, use of a validated tool to assess the DII and detailed assessment of obesity-related parameters. Some limitations of the current study are essential to be acknowledged. First, the original DII developed by Shivappa *et al* 19 is based on intakes of 45 food parameters, while, in the present study, the DII was calculated based on 37 dietary parameters available in the FFQ. Second, dietary intake was measured using a validated FFQ that is prone to memory bias. Third, in addition to the FTO gene, there are some SNPs in other genes that their relationships with obesity indices are demonstrated, but gene–gene interactions were not evaluated in this study. Finally, the follow-up period of this study was relatively short, which might have resulted in inadequacy of long-term outcomes.

## Conclusion

In conclusion, the present study for the first time indicated that there may be an interactive effect between inflammatory potential of diet and the FTO rs1421085genotypes on change in WC. Moreover, higher score for DII was significantly related to elevated odds of having high VAI in individuals with wild-type homozygous genotype of FTO SNPs; indeed, these people are more susceptible to have visceral adiposity with high DII diets. These data also emphasise that individuals with the A allele of rs8050136 in FTO following a diet with higher DII are susceptible to higher WC, but lower WHR. Therefore, paying attention to gene–diet interactions can deliver personalised recommendations to achieve a better status in prevention and management of obesity. This study proposes that people should be genotyped before delivering personalised dietary suggestions. Further well-designed studies are required in different racial populations to elucidate the biology of FTO SNPs and their impacts on the relation of DII to obesity phenotypes.

## Data Availability

Data are available on reasonable request.
