# Supplementary material for: Interaction of common variants of FTO gene and Dietary Inflammatory Index on obesity measures: Tehran Lipid and Glucose Study
Source: BMJ Nutr Prev Health. 2023 Nov 23;6(2):332–40. doi: 10.1136/bmjnph-2023-000665 (PMC11009517; doi:10.1136/bmjnph-2023-000665)
Supplement: Supplementary data [file bmjnph-2023-000665supp001.pdf]

Supplementary Table 1. Linear regression analysis for the association of anthropometric indices at baseline with dietary inflammatory index score based on FTO SNP genotypes

	BMI [a]		WC [a]		WHR [a]		VAI [a]	
	β	P	β	P	β	P	β	P
**rs1121980**								
CC	-0.02	0.57	-0.02	0.3	-0.005	0.81	-0.02	0.35
CT + TT	-0.1	<0.001	-0.08	<0.001	-0.04	0.01	-0.06	0.001
P interaction	0.02		0.001		0.05		0.14	
**rs1421085**								
TT	-0.02	0.46	-0.03	0.26	-0.006	0.77	-0.02	0.51
TC + CC	-0.1	<0.001	-0.08	<0.001	-0.04	0.01	-0.07	0.001
P interaction	<0.001		<0.001		0.12		0.07	
**rs8050136**								
GG	-0.03	0.16	-0.04	0.08	-0.01	0.50	-0.01	0.56
GA + AA	-0.1	<0.001	-0.08	<0.001	-0.04	0.03	-0.07	0.001
P interaction	<0.001		0.002		0.08		0.22	
**GRS**								
GRS<2.83	-0.03	0.18	-0.04	0.11	-0.01	0.47	-0.01	0.62
GRS≥2.83	-0.1	<0.001	-0.09	<0.001	-0.04	0.02	-0.07	0.002
P interaction	<0.001		0.001		0.08		0.23	

[a]Body mass index (BMI), waist circumference (WC), Waist-to-hip ratio (WHR) and visceral adiposity index (VAI) at baseline

FTO: Fat mass and Obesity associated gene, SNP: Single Nucleotide Polymorphism. Standardized β were calculated using linear regression model, adjusted for education level, age, gender, smoking status, physical activity and energy intake. Participants were classified (8 groups) according to quartiles of dietary inflammatory index and dominant model of FTO polymorphism genotypes or genetic risk score (GRS) ≥ median and <median. P interaction was calculated using General Linear Model.
